# Reply to: Comment on “Self-organizing neuromorphic nanowire networks as stochastic dynamical systems”

**DOI:** 10.1038/s41467-026-77956-5

**Published:** 2026-11-09

**Authors:** Gianluca Milano, Fabio Michieletti, Davide Pilati, Carlo Ricciardi, Enrique Miranda

**Affiliations:** 1 Advanced Materials Metrology and Life Sciences Division, INRiM (Istituto Nazionale di Ricerca Metrologica), Torino, Italy; 2 Department of Applied Science and Technology, Politecnico di Torino, Torino, Italy; 3 Departament d’Enginyeria Electrònica, Universitat Autònoma de Barcelona (UAB), Cerdanyola del Vallès, Spain

**Keywords:** Electrical and electronic engineering, Electronic devices, Complex networks

In our work^1^, we developed a compact modeling framework describing the dynamics of self-organizing memristive nanowire networks using an Itô-type stochastic differential equation that combines deterministic and stochastic contributions within a single state equation with memory. The model was designed to support efficient in silico investigations of the computational capabilities of these systems within the reservoir computing paradigm, while maintaining the mathematical simplicity required for implementation in circuit simulators. Rather than reproducing every aspect of the underlying microscopic dynamics, it provides a unified phenomenological description of the experimentally relevant observables by retaining the essential physical mechanisms governing the device behavior. This reduced-order representation enables computational efficiency and analytical tractability, making large-scale circuit-level simulations and systematic exploration of unconventional computing strategies feasible. As with any compact model, this level of abstraction may, under certain operating conditions, lead to quantitative deviations from the underlying physical dynamics; however, it preserves the key phenomenology relevant to the intended computational applications.

Our modeling approach assumes that the conductance dynamics are governed by a single dominant physical process, resulting in an exponential relaxation behavior. Such relaxation is characterized by a single intrinsic timescale, whereas the power-law description discussed by the authors of the Comment is scale-free and therefore does not possess a characteristic timescale, giving rise to long-term memory effects. A key advantage of our approach is that it allows the control parameter, namely the applied voltage, to be incorporated directly into a standard first-order differential equation. This provides a unified framework in which the conductance dynamics and their voltage dependence are naturally coupled. Consequently, the model can describe the time evolution of the system under arbitrary excitation signals while remaining fully consistent with Chua’s formulation of memristive systems. This makes the proposed approach particularly suitable for compact device modeling and efficient circuit-level simulation using standard SPICE-based tools.

We modeled conductance jumps using a homogeneous Poisson process with a constant event rate, thereby assuming statistically independent switching events and neglecting temporal correlations as a first-order approximation. A more complete description of the inter-event interval (IEI) statistics would require power-law rather than exponential distributions. Indeed, in our previous work we showed that, under specific operating conditions, the system exhibits scale-free event statistics and signatures of self-organized criticality^2^. Nevertheless, the aim of the present work is not to provide a complete statistical description of the switching dynamics, but rather to develop a compact model that captures their essential phenomenology while remaining compatible with efficient circuit simulation. Within this framework, the homogeneous Poisson approximation offers a simple, computationally efficient, and physically meaningful mechanism for generating stochastic switching events that reproduce the key experimentally observed features.

We approximate the system dynamics using an Ornstein-Uhlenbeck process, assuming that the continuous noise and discrete conductance jumps are statistically independent. This approximation neglects possible feedback or coupling between these stochastic processes, which may arise under certain experimental conditions. Incorporating such interactions would require a substantially more sophisticated stochastic formulation that introduces additional model parameters and significantly increases the computational cost, thereby compromising the simplicity and efficiency that are central to the compact modeling philosophy.

Accordingly, the proposed model was conceived as a computationally efficient phenomenological representation rather than as a complete microscopic description of the underlying physical mechanisms. While the framework discussed in the Comment is primarily intended to provide a more detailed account of the device physics, our objective is different: to develop a compact model suitable for efficient circuit simulation and for investigating the computational capabilities of these memristive systems. In this context, compact models complement, rather than replace, detailed physical descriptions. By retaining the essential dynamical features required to reproduce the experimentally observed behavior, they enable circuit-level simulations and large-scale computational studies that would otherwise be computationally prohibitive.

We thank the authors for their thoughtful analysis, which offers valuable insight into the emergent behavior of self-organizing memristive systems. Our decision to make the experimental datasets openly available was guided by the aim of encouraging scientific dialog across materials science, stochastic processes, and the physics of dynamical systems. We believe that the interplay between complementary modeling approaches and experimental observations is essential for advancing the understanding of these complex systems and for developing progressively more accurate and physically grounded models.
